# Surface-Engineered Mitochondria with Targeting Potential for Endothelial Repair

**DOI:** 10.1007/s12195-025-00862-1

**Published:** 2025-08-22

**Authors:** Brandon Applewhite, Natalia Matiuto, Aurea del Carmen, Bin Jiang

**Affiliations:** 1 Center for Advanced Regenerative Engineering, Northwestern University, Evanston, IL USA; 2 Department of Biomedical Engineering, Northwestern University, Chicago, IL USA; 3 Department of Surgery, Northwestern University Feinberg School of Medicine, Chicago, IL USA; 4 Querrey Simpson Institute for Regenerative Engineering, Northwestern University, Chicago, IL USA

**Keywords:** Mitochondrial transplantation, Surface engineering, Endothelial dysfunction, Lipid-polymer coatings, Vascular regeneration, Bioenergetic restoration

## Abstract

**Purpose:**

Mitochondrial dysfunction contributes to endothelial injury in vascular diseases and interventions. While mitochondrial transplantation offers a promising therapeutic strategy, current approaches lack target specificity, efficient uptake, and long-term retention. This study presents a surface-engineering approach to enhance mitochondria delivery to the vascular endothelium as a step toward novel endothelial repair strategies.

**Methods:**

Mitochondria were isolated from healthy induced pluripotent stem cell-derived mesenchymal stem cells (iPSC-MSCs) and surface functionalized with a phospholipid-based coating platform (DSPE-PEG) to enable peptide functionalization. DSPE-PEG was conjugated to either VCAM-1-binding peptide and collagen-binding peptide to enable targeting to dysfunctional and injured endothelium. Mitochondria particle characteristics were measured using flow cytometry, dynamic light scattering and Seahorse. Mitochondrial uptake, retention, and function were assessed in human diabetic aortic endothelial cells (DAECs) using confocal microscopy, flow cytometry, JC-1 staining, and Seahorse metabolic analysis.

**Results:**

iPSC-MSCs provided bioenergetically competent mitochondria suitable for therapeutic delivery. DSPE-PEG surface functionalization significantly enhanced mitochondrial uptake in DAECs, compared to uncoated mitochondria. Confocal imaging and quantitative analysis revealed increased cytoplasmic retention and greater colocalization with the endogenous mitochondrial network after 24 h. Functional assays demonstrated improved mitochondrial membrane potential and sustained oxygen consumption in recipient cells, indicating enhanced host mitochondrial function following treatment with surface-engineered mitochondria.

**Conclusions:**

This study establishes a proof-of-concept for mitochondria surface engineering to enhance mitochondria transplantation to damaged endothelium, demonstrating improved cellular uptake and bioenergetic restoration. These findings provide a foundation for developing adaptable, cell-free therapeutics for vascular disease.

**Supplementary Information:**

The online version contains supplementary material available at 10.1007/s12195-025-00862-1.

## Introduction

Mitochondria, the powerhouse of eukaryotic cells, are responsible for many essential cellular functions, including energy metabolism, calcium homeostasis, cell survival, differentiation, and death. Dysfunctional mitochondria contribute to a wide range of diseases, including cardiovascular diseases (CVDs), neurodegenerative disorders, metabolic syndromes, and aging-related conditions [1]. Therapeutic strategies aimed at restoring mitochondrial function hold immense potential across multiple biomedical fields. Mitochondrial transplantation has gained increasing attention as a potential regenerative strategy for rejuvenating damaged cells and tissues [2–4]. Various studies have demonstrated that exogenous mitochondria can integrate into recipient cells, restoring bioenergetic function, reducing oxidative stress, and improving survival [5–8]. Despite this promise, challenges remain in delivering mitochondria efficiently, maintaining their viability, and ensuring targeted uptake by specific cell types.

Advances in bioengineering and nanomedicine have revolutionized the field of therapeutic delivery by enabling precise control over biomaterial interactions at the molecular level. By leveraging approaches such as surface functionalization, biomimetic coatings, and ligand-directed targeting, researchers have successfully enhanced stability, circulation time, and cell-specific uptake of therapeutic cargo, ranging from nanoparticles to gene therapies [9]. These principles can be extended to mitochondrial transplantation, addressing critical challenges currently experienced during mitochondrial delivery. One promising strategy to overcome these hurdles is the surface engineering of mitochondria using poly(ethylene glycol)–distearoylphosphatidylethanolamine (DSPE-PEG) block copolymer coatings and bioactive peptides. Inspired by extracellular vesicles, lipid nanoparticles, and synthetic nanocarriers, these modifications may allow mitochondria to evade immune clearance, prevent premature degradation, and preserve bioenergetic function and membrane polarization [10], 11. These coatings also provide a versatile platform for incorporating cell-binding ligands, enabling controllable interactions with target cells and tissues.

In this study, we engineered a DSPE-PEG–based coating platform to enhance mitochondrial delivery to the vascular endothelium. As the innermost lining of blood vessels, the endothelium plays a central role in maintaining vascular homeostasis, and its dysfunction—whether due to chronic disease, inflammation, or surgical injury—is tightly linked to mitochondrial impairment. Chronic endothelial dysfunction, characterized by oxidative stress and reduced nitric oxide bioavailability, is a hallmark of atherosclerosis, hypertension, and diabetes-associated vascular disease and is associated with an impairment in mitochondria function [12–15]. Additionally, both endovascular procedures (e.g., angioplasty and stenting) and open vascular surgeries (e.g., bypass grafting) cause direct or ischemia-reperfusion–induced damage to the endothelium, disrupting mitochondrial membrane potential and impairing oxidative phosphorylation [16]. Endothelial mitochondrial damage contributes to oxidative stress, apoptosis, and barrier breakdown, exacerbating vascular inflammation and remodelling [17]. Therefore, restoring mitochondrial function in dysfunctional or injured endothelium presents a promising therapeutic strategy for both chronic and acute vascular conditions.

To facilitate selective endothelial uptake, we functionalized isolated mitochondria with DSPE-PEG conjugated to peptides targeting (1) VCAM-1, an inflammatory adhesion molecule upregulated in chronic vascular diseases [18], and (2) collagen IV, a subendothelial matrix protein exposed upon endothelial injury [19–21]. We systematically evaluated how this surface functionalization influences mitochondrial stability, uptake, and integration, as well as endothelial bioenergetics. As an initial disease-relevant model, we used human diabetic aortic endothelial cells (DAECs), which exhibit baseline mitochondrial dysfunction due to their diabetic origin and thus serve as a useful platform to assess therapeutic mitochondrial delivery [22]. While this in vitro model does not fully replicate the complexity of inflamed or injured endothelium in vivo, it provides a consistent and relevant system to test the performance of engineered mitochondria. Together, this work lays the foundation for precision mitochondrial delivery technologies with therapeutic potential in both chronic and acute vascular injury contexts.

## Methods

### Mesenchymal Stem Cell Differentiation from Induced Pluripotent Stem Cells

Induced pluripotent stem cells (iPSCs) were reprogrammed from blood mononuclear cells from a young, healthy male (XY) donor (N = 1 biological replicate) as previously described [23, 24]. Mesenchymal stem cells (MSCs) were differentiated from IPSC using STEMdiff^tm^ Mesenchymal Progenitor Kit (Stemcell Technologies) according to the manufacturer’s protocol. MSC phenotype was confirmed after 21 days with immunocytochemistry and flow cytometry for positive expression of canonical MSC markers CD73, CD90, CD105 and negative expression of pluripotent stem cell marker SSEA4 (MSC Characterization Panel, Stemcell Technologies). Differentiated MSCs were grown in Mesenchymal Stem Cell Growth Media (Lonza, Walkersville, MD).

### Mitochondria Isolation From iPSC-MSCs

Mitochondria were isolated from iPSC-MSCs using a cell lysis method (Mitochondria Isolation Kit, Thermo Fisher Scientific) using approximately 15x10^6 cells. Cells were dissociated with trypsin, and resuspended in 800 µl of buffer A with 10 µl of Mitochondria Isolation Reagent B. Cells were incubated on ice for 5 min, vortexing every minute, before adding 800 uL of Reagent C and centrifugation at 700×*g* for 10 min at 4 °C. The supernatant was transferred to a new tube and centrifuged at 3000×*g* for 15 min at 4 °C. The isolated mitochondrial pellet was resuspended in 500 µl Reagent C with and then centrifuged at 12000×*g* for final purification. After isolation mitochondria were resuspended in 1 mL of Reagent C buffer (Mitochondria Isolation Kit, Thermo Fisher Scienfitic) and protein content was quantified using the BCA assay. To visualize mitochondria in downstream experiments, detached cells were incubated in media containing 500 nM Mitotracker CmxRos (Thermo Fisher Scientific) for 45 min at 37 °C before isolating mitochondria. Multiple batches of mitochondria from different isolations were used for this study.

### Polymer Peptide Conjugate Synthesis and Characterization

Biotinylated VCAM-1 binding peptide (VBP: VHPKQHRGGSKGC) [25] and biotinylated collagen binding peptide (CBP: CQDSETRTFY)[26]were custom ordered from ABI Scientifics (Sterling, VA). The peptides were reacted with 1,2-distearoyl-sn-glycero-3-phosphoethanolamine-N-[maleimide(polyethylene glycol)− 5000] (DSPE-PEG-MAL, Nanosoft Polymers, Winston-Salem, NC) in ultrapure water at a thiol:maleimide molar equivalent at room temperature for 24 h to yield DSPE-PEG-VBP and DSPE-PEG-CBP conjugates. The reaction products were purified by dialysis for 24 h with a Slide-a-Lyzer Dialysis Cassette (MWCO 7,000 kDa, ThermoFisher) before lyophilization. Successful conjugation was confirmed using MALDi mass spectrometry by the Northwestern Integrated Molecular Structure Education and Research Center.

### Collagen Binding Assay

To assess the binding capacity of CBP after DSPE-PEG conjugation, increasing concentrations of biotinylated CBP (CBP-biotin) and DSPE-PEG-CBP-biotin were added to either collagen I (10 ug/cm^2^) coated plates or Millicoat® Human Collagen Type IV Coated Strips (Sigma Aldrich) for 3 h at 37 °C. Plates were then rinsed with PBS to remove unbound CBP and DSPE-PEG-CBP and incubated with streptavidin-594 (4ug/ml) for 30 min before rinsing and measuring fluorescence with a plate reader.

### Surface Engineering of Isolated Mitochondria

To modify the surface of isolated mitochondria, aliquots of freshly isolated mitochondria were combined with solutions of DSPE-PEG-peptide conjugate (1 mg/mL in Reagent C) at increasing mass ratios of polymer to mitochondria protein in Reagent C buffer and incubated for 3 h on ice while shaking. After incubation, functionalized mitochondria were rinsed by centrifugating at 12,000×*g* for 5 min and replacing the supernatant with fresh Reagent C twice before resuspending in Mitochondria Storage Buffer (Mitochondria Isolation Kit, Millipore Sigma) and storing at 4 °C for immediate studies or − 80 °C for long term (<14 days) storage. Multiple batches were used for downstream experiments.

### Flow Cytometry Analysis of Coating Efficiency

Coating efficiency was determined by flow cytometry by incubating Mitotracker labeled DSPE-PEG-Peptide coated mitochondria with AlexaFluor-488 streptavidin (1:250) for 45 min to allow reaction with the biotin group conjugated to the peptide. After 45 min, mitochondria were rinsed with Reagent C buffer and analyzed by flow cytometry. Coating efficiency was calculated from the ratio of Mitotracker Red, AlexaFluor-488 double positive particles to total Mitotracker Red positive particles. Uncoated mitochondria were used as a negative control. Confocal microscopy was used to visually confirm mitochondria coating.

### Particle Size Analysis

Mitochondria particle size and zeta potential was measured with a Zetasizer Nano ZSP (Malvern Instruments). Mitochondria were dispersed in MilliQ water and loaded into Malvern Panalytical Folded Capillary Zeta Cells (Malvern Instruments). Particle size distributions were averaged across 3 runs (n = 3).

### Mitochondria Uptake in Diabetic Human Aortic Endothelial Cells

The effect of DSPE-PEG and DSPE-PEG-peptide surface coating on mitochondria uptake was assessed using Diabetic Human Aortic Endothelial Cells (DAECs) (Lonza) known to have increased expression of VCAM-1 [22]. Cells were seeded at 10,000 cells/chamber in Nunc™ Lab-Tek™ II Chambered Coverglass and labeled with Mitotracker Deep Red (Thermo Fisher Scientific) to visualize endogenous mitochondria. After 24 h, uncoated, DSPE-PEG coated and DSPE-PEG-VBP functionalized mitochondria labeled with Mitotracker Red Cmxros were added at 1 μg/2000 cells. Mitochondria-containing media was removed after 24 h and cells were rinsed with phosphate buffered saline (PBS) before fixing with paraformaldehyde. Fixed cells were stained with phalloidin-488 for 45 min and nuclei were counterstained with Hoechst. To assess the fate of mitochondria after internalization, a similar method was used except media was replaced with endothelial cell growth media (EGM-2, Lonza) after 24 h. DAECs were then fixed 72 h after mitochondria coculture. Internalization was visualized using a spinning disk confocal microscope (Nikon SORA). The mitochondria area per cell and the size of exogenous mitochondria particles were calculated from thresholded 60x images of individual cells using the Fiji Mitochondria Analyzer Plugin. For size analysis, the 100 largest particles were used for analysis. Colocalization of internalized mitochondria with the endogenous mitochondria was quantified using the JaCoP plugin and the Manders’ coefficients were reported [26].

### Seahorse Assay for Cells

The effect of artificial mitochondria transfer on DAECs oxidative phosphorylation was measured using the Seahorse XF Cell Mito Stress Test Kit on a Seahorse XF96 Analyzer (Agilent). DAECs (passage 4-8) were seeded at 20,000 cells/well on Seahorse XFe96/XF Pro Cell Culture Microplates. After 24 h, uncoated and DSPE-PEG coated mitochondria were added at 1 μg/2000 cells and incubated for 24 h. Wells were then rinsed with PBS before changing to Seahorse medium (XF DMEM medium, pH 7.4, 1 mM pyruvate, 2 mM glutamine, 10 mM glucose). To measure the lasting effects on cell respiration, mitochondria-containing media was replaced with EGM-2 and cells were grown another 72 h before changing to Seahorse medium. Final well concentrations of oligomycin, carbonyl cyanide−4 (trifluormethoxy) phenylhydrazone (FCCP), and rotenone/antimycin A were 1.5, 1.0, and 0.5 μM respectively. A similar protocol was used to measure the respiratory function of IPSC-MSCs except cells were seeded overnight at 15,000 cells/well without mitochondria treatment, and the assay was performed the next day. Spare respiratory capacity and coupling efficiency were calculated using a standard method [27]. Specifically, coupling efficiency was calculated as:

$$\text{Coupling } \text{efficiency }\left(\%\right)=\frac{\text{ATP}-\text{linked OCR}}{\text{Basal OCR}}\times 100\%$$

where ATP-linked OCR = Basal OCR – Oligomycin-inhibited OCR (State IV).

Spare respiratory capacity was calculated as:

$$\text{Spare} \text{respiratory capacity }(\%)=\frac{\text{Maximal} \text{OCR}- \text{Basal OCR}}{\text{Basal OCR}}\times 100\%$$

where maximal OCR was determined after FCCP injection.

### Seahorse Assay on Isolated Mitochondria

The bioenergetic activity of isolated mitochondria after storage was assessed using the Seahorse XF Cell Mito Stress Test Kit as preciously described [28]. Briefly, frozen mitochondria were thawed on ice and added to a Seahorse XFe96/XF Pro Cell Culture Microplates at 10 ug/well. The plate was centrifuged at 2,000 g for 20 min at 4 to ensure mitochondria adhered to the plate. After centrifugation, mitochondria respiration buffer (220 mM mannitol, 70 mM sucrose, 10 mM potassium phosphate, 5 mM magnesium chloride, 1 mM ethylene glycol-bis(2-aminoethylether)-N,N,N′,N′-tetraacetic acid), 2 mM HEPES, 10 mM glutamate, 10 mM malate in deionized water) with adenosine diphosphate (20 mM) was added to the wells to bring the final volume to 180 uL/well. Final port concentrations of oligomycin, FCCP, and rotenone/antimycin A were 5 μM, 20 μM, and 5 μM, respectively.

### JC-1 Staining

To assess mitochondrial membrane potential (Δψm), DAECs were incubated with JC-1 dye (1 µg/mL) in complete media for 30 min at 37 °C. JC-1 is a cationic dye that accumulates in mitochondria in a membrane potential–dependent manner [29]. In healthy, polarized mitochondria, JC-1 forms aggregates that emit red fluorescence (emission ~ 590 nm); in depolarized or dysfunctional mitochondria, the dye remains in its monomeric form and emits green fluorescence (emission ~ 529 nm). Following incubation, cells were washed and imaged immediately using an EVOS microscope.

### Statistical Analysis

All statistical analyses were performed using GraphPad Prism 10.4 software (GraphPad Software, La Jolla, CA). Comparisons between multiple groups were performed by Brown-Forsythe and Welch Anova tests unless otherwise specified. Unpaired student’s t-test or Mann-Whitney test was used for comparisons between two groups. Two way ANOVA was used for comparisons across multiple timepoints. A value of p < 0.05 was considered to be statistically significant.

## Results

### iPSC-Derived MSCs Serve as a Reliable Source of Functional Mitochondria

**Fig. 1:**
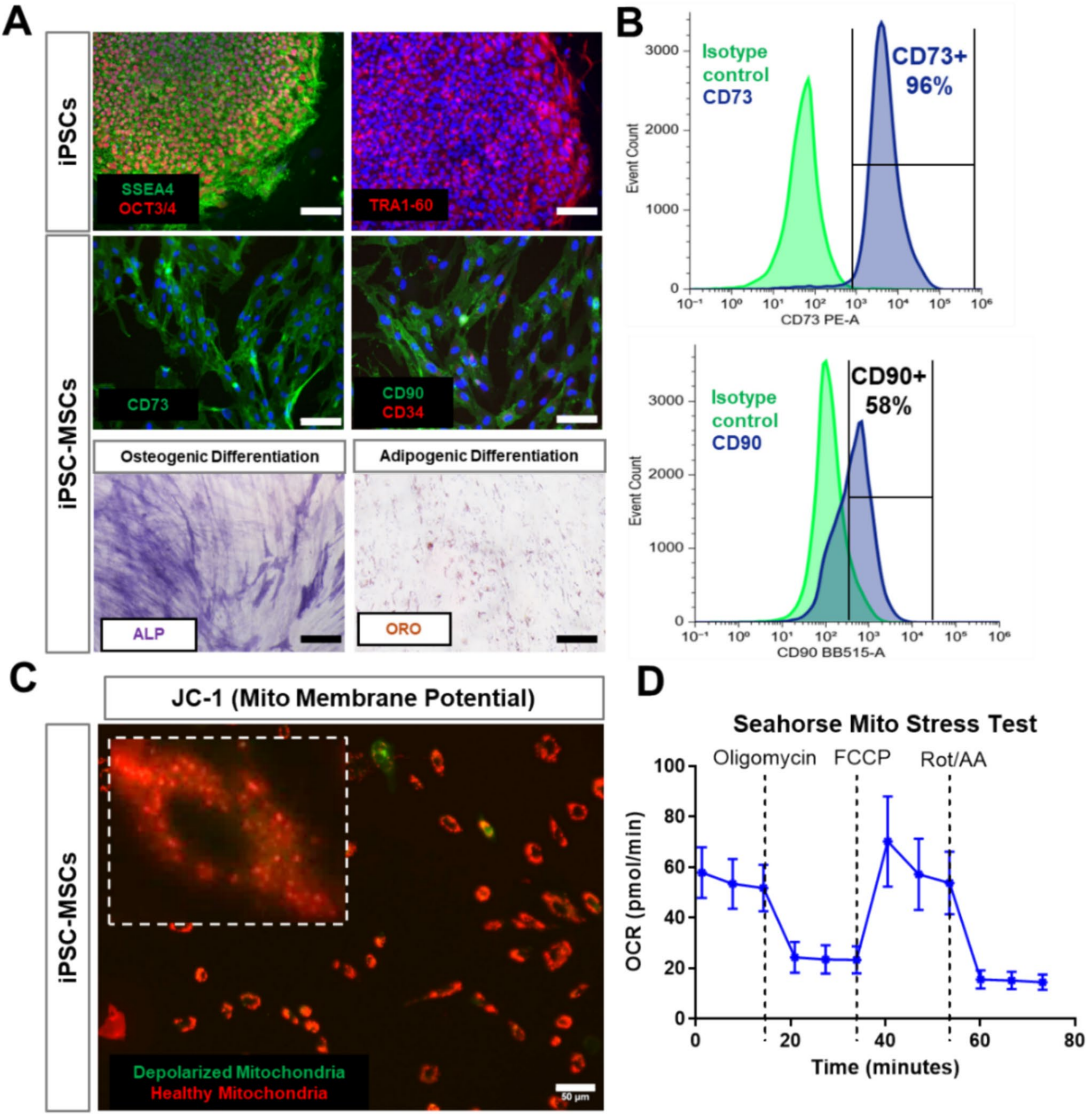
Characterization of iPSC-MSCs as a Source of Functional Mitochondria. **A** Immunofluorescence staining of iPSCs and iPSC-MSCs with their phenotypic markers and differentiation potential of iPSC-MSCs for osteogenesis (ALP positive) and adipogenesis (ORO positive). Scale bar = 100 µm. **B** Flow cytometry analysis of iPSC-MSC surface markers CD73 and CD90. **C** JC-1 staining of mitochondrial membrane potential in iPSC-MSCs. A predominant red fluorescence signal indicates high mitochondrial membrane potential, confirming the presence of functionally polarized mitochondria. Scale bar = 50 µm. **D** Seahorse extracellular flux analysis of iPSC-MSCs (n = 3). Oxygen consumption rate (OCR) curve demonstrates normal respiratory function, confirming that iPSC-MSCs maintain bioenergetically active mitochondria.

To generate a reliable and scalable source of functional mitochondria for therapeutic applications, we differentiated MSCs from iPSCs reprogrammed from blood mononuclear cells of a healthy young adult male. The iPSCs were previously reprogrammed in-house and underwent differentiation following a commercially established protocol [23]. Characterization of iPSCs and iPSC-derived MSCs (iPSC-MSCs) was performed using immunofluorescence staining for phenotypic markers (Fig. 1A). iPSCs exhibited strong expression of SSEA4, OCT3/4, and TRA-1-60, confirming their pluripotency. Following differentiation, iPSC-MSCs expressed CD73 and CD90, characteristic markers of MSCs [30], while showing no detectable expression of CD34, a hematopoietic marker. The multipotency of iPSC-MSCs was further validated by their ability to undergo osteogenic and adipogenic differentiation, demonstrated by alkaline phosphatase (ALP) staining for osteogenesis and oil red O staining for adipogenesis (Fig. 1A). Flow cytometry analysis further confirmed high differentiation efficiency, with a strong population of CD73+ and CD90+ cells (Fig. 1B). To ensure that the iPSC-MSCs harbored functional and bioenergetically active mitochondria, we evaluated mitochondrial membrane potential and respiratory function. JC-1 staining revealed a predominant red fluorescence signal, indicating a high mitochondrial membrane potential and the presence of healthy, polarized mitochondria in iPSC-MSCs (Fig. 1C). Additionally, Seahorse metabolic analysis demonstrated a normal respiratory profile, with a high coupling efficiency (76.7 ± 4 %) and spare respiratory capacity (147.3 ± 18.2 %) supporting the presence of metabolically active mitochondria (Fig. 1D).

### DSPE-PEG-Peptide Conjugates Enable Surface Engineering of Isolated Mitochondria

**Fig. 2:**
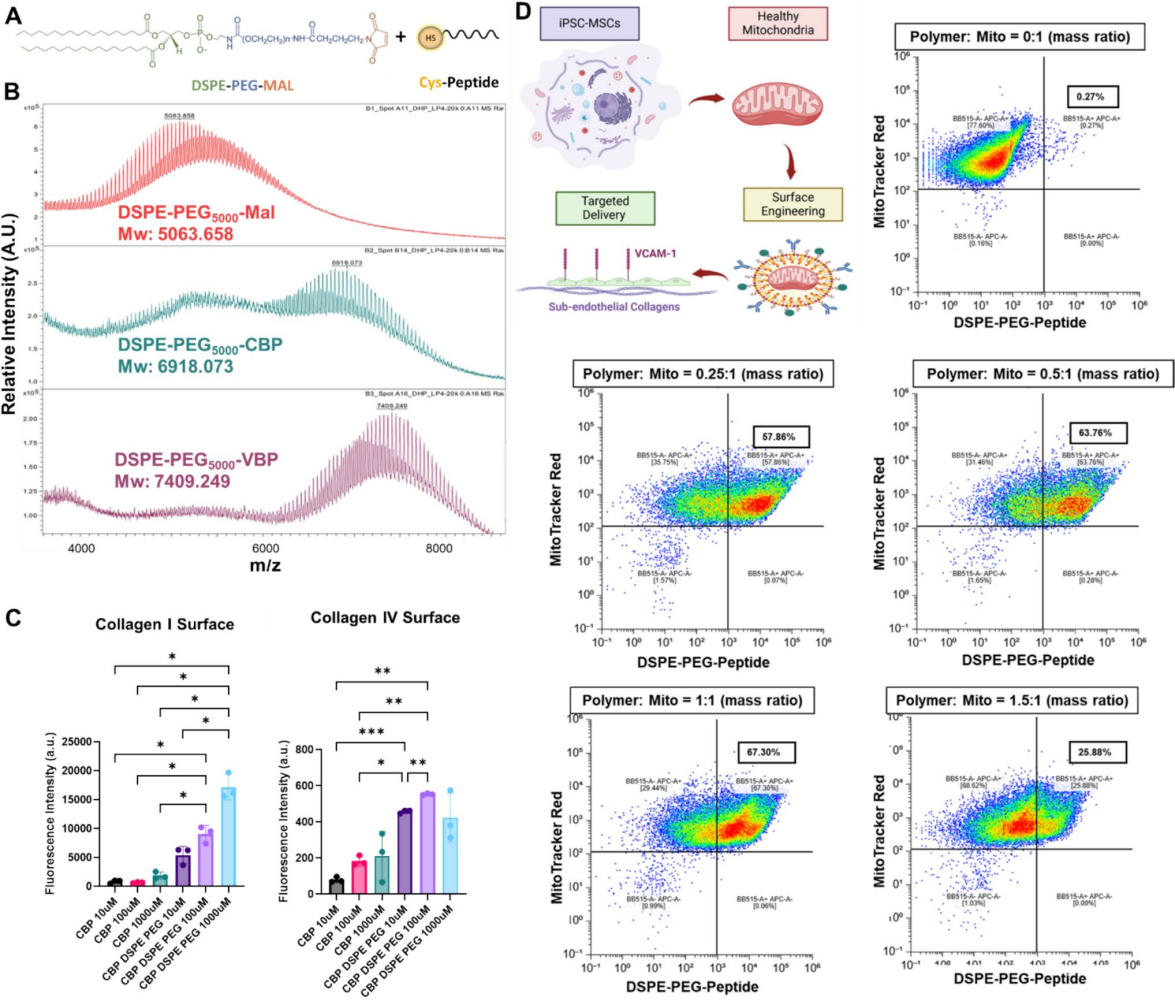
Surface Engineering of Mitochondria with DSPE-PEG-Peptide Conjugates for Targeted Endothelial Delivery. **A** Schematic representation of the maleimide-thiol conjugation reaction used to attach peptides to DSPE-PEG-MAL for mitochondrial surface functionalization. Peptides were biotinylated to enable characterization via fluorescence-labeled streptavidin. **B** MALDI mass spectrometry confirmed successful conjugation, with molecular weight shifts corresponding to DSPE-PEG-MAL, DSPE-PEG-CBP, and DSPE-PEG-VBP, verifying efficient peptide attachment. **C** Functional binding assay demonstrating significantly enhanced affinity of DSPE-PEG-CBP to collagens I and IV, compared to unconjugated CBP (* = p < 0.05, ** = p < 0.01, *** = p < 0.005, Brown-Forsythe and Welch ANOVA). **D** Schematic representation of mitochondria surface engineering with DSPE-PEG-peptide conjugates. **E** Flow cytometry analysis of DSPE-PEG-peptide attachment to isolated mitochondria at various mass ratios.

To enable facile peptide functionalization of isolated mitochondria, we utilized DSPE-PEG-peptide conjugates to modify the mitochondria surface. Specifically, we conjugated CBP to recognize exposed subendothelial collagens following vascular injury and VBP to target VCAM-1 which becomes upregulated during vascular inflammation as a proof-of-concept for future applications of targeted mitochondria delivery. The conjugation process via maleimide-thiol chemistry is illustrated in the schematic (Fig. 2A). Successful conjugation of CBP and VBP to DSPE-PEG-MAL was confirmed by MALDI mass spectrometry, with molecular weight shifts corresponding to DSPE-PEG-MAL, DSPE-PEG-CBP, and DSPE-PEG-VBP, verifying efficient peptide attachment (Fig. 2B). To assess functional binding, the binding affinity of DSPE-PEG-CBP to collagens I and IV was compared to CBP which has previously been shown to have high binding affinity to both substrates [31]. Both CBP and DSPE-PEG-CBP exhibited a dose-dependent increase in binding, however DSPE-PEG-CBP significantly enhanced binding capability compared to the unconjugated peptide (Fig. 2C), supporting its potential for targeted endothelial delivery. Following conjugation, we optimized polymer coating efficiency on isolated mitochondria by incubating mitochondria isolated from iPSC-MSCs with biotinylated DSPE-PEG-peptide at varying ratios (Fig. 2D) and assessing successful surface modification via flow cytometry. With increasing polymer concentrations, the percentage of mitochondria positive for polymer attachment increased, reaching a peak of 67% at a 1:1 mitochondrial-to-polymer mass ratio (Fig. 2E). However, further increasing the polymer amount to 1.5:1 resulted in a decrease in polymer signal, suggesting potential steric hindrance or polymer aggregation effects.

### DSPE-PEG-Peptide Surface Engineering Alters Mitochondria Particle Physical Properties and Improves Respiration

**Fig. 3:**
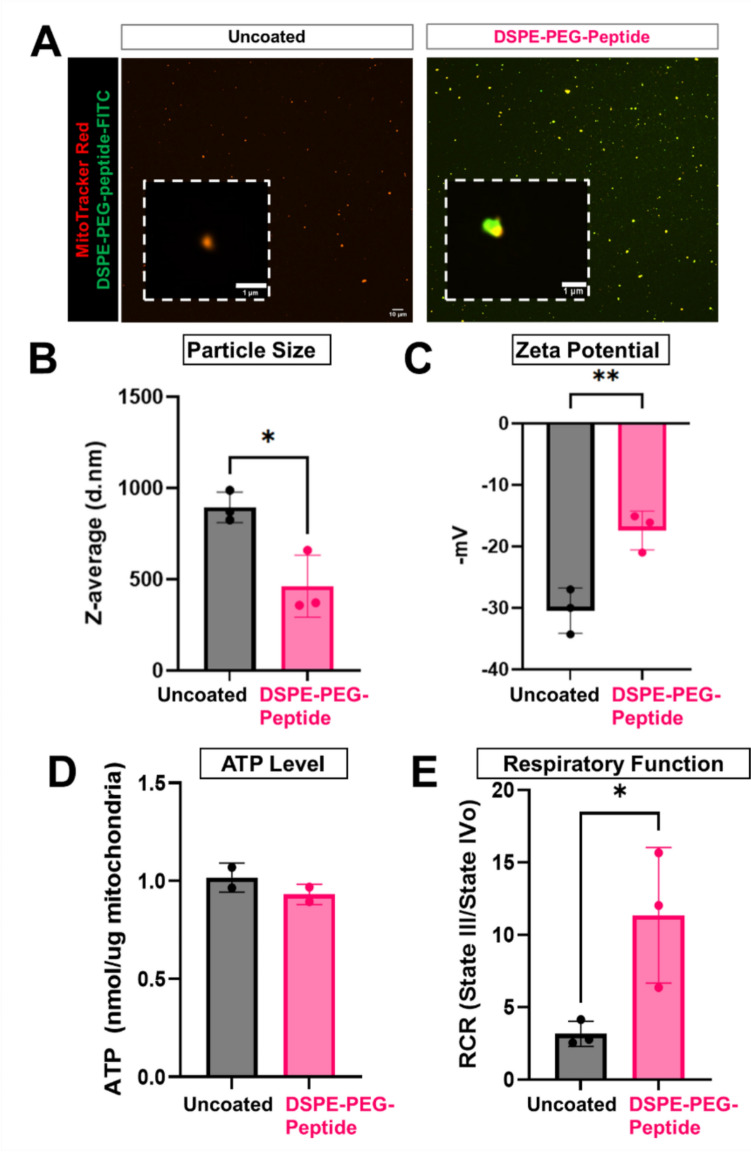
Characterization of optimized polymer coated mitochondria. **A** Confocal microscopy showing colocalization of MitoTracker Red-labeled mitochondria (red) and FITC-labeled DSPE-PEG-peptide (green). Scale bar = 1 µm. **B** Particle size distribution analysis showing reduced average diameter after surface coating. **C** Zeta potential measurements indicating decreased negative surface charge following peptide coating. **D** ATP content of isolated mitochondria remained stable after coating. **E** Respiratory control ratio (RCR) calculated from Seahorse XF Mito Stress Test, showing a significant increase in coupling efficiency in coated mitochondria. (n = 3; *p < 0.05, **p < 0.01 by unpaired two-tailed Student’s t-test.)

To evaluate the effects of DSPE-PEG-peptide coating on mitochondrial properties, we characterized the morphology, size distribution, surface charge, and metabolic activity of DSPE-PEG-peptide coated (at 1:1 mass ratio) and uncoated mitochondria. Fluorescent microscopy showed that uncoated mitochondria, labeled with MitoTracker Red, exhibited red fluorescence only, whereas DSPE-PEG–peptide–modified mitochondria displayed both red and green fluorescence after incubation with FITC-streptavidin, indicating successful surface coating (Fig. 3A). Dynamic light scattering analysis of mitochondria particles revealed DSPE-PEG-peptide modification reduces the average particle diameter compared to uncoated mitochondria (Fig. 3B, Supplementary Fig. S1). Zeta potential measurements showed that both uncoated and polymer-coated mitochondria carried a net negative charge, but polymer-coated mitochondria exhibited a less negative zeta potential, closer to neutrality (Fig. 3C), suggesting surface modification alters the electrostatic properties of mitochondria which can influence their interactions with target cells. To assess mitochondrial metabolic competence following surface engineering, we measured ATP levels and performed a Seahorse XF Mito Stress Test on freshly isolated mitochondria (Supplementary Fig. S1). ATP quantification showed no significant difference between coated and uncoated groups, confirming preservation of basal bioenergetic capacity (Fig. 3D). Notably, surface-coated mitochondria exhibited a significantly higher respiratory control ratio (state III/state IV oxygen consumption rate), indicating improved coupling efficiency and enhanced respiratory potential compared to uncoated mitochondria (Fig. 3E).

### Surface Functionalization Enhances Endothelial Uptake of Exogenous Mitochondria

**Fig. 4:**
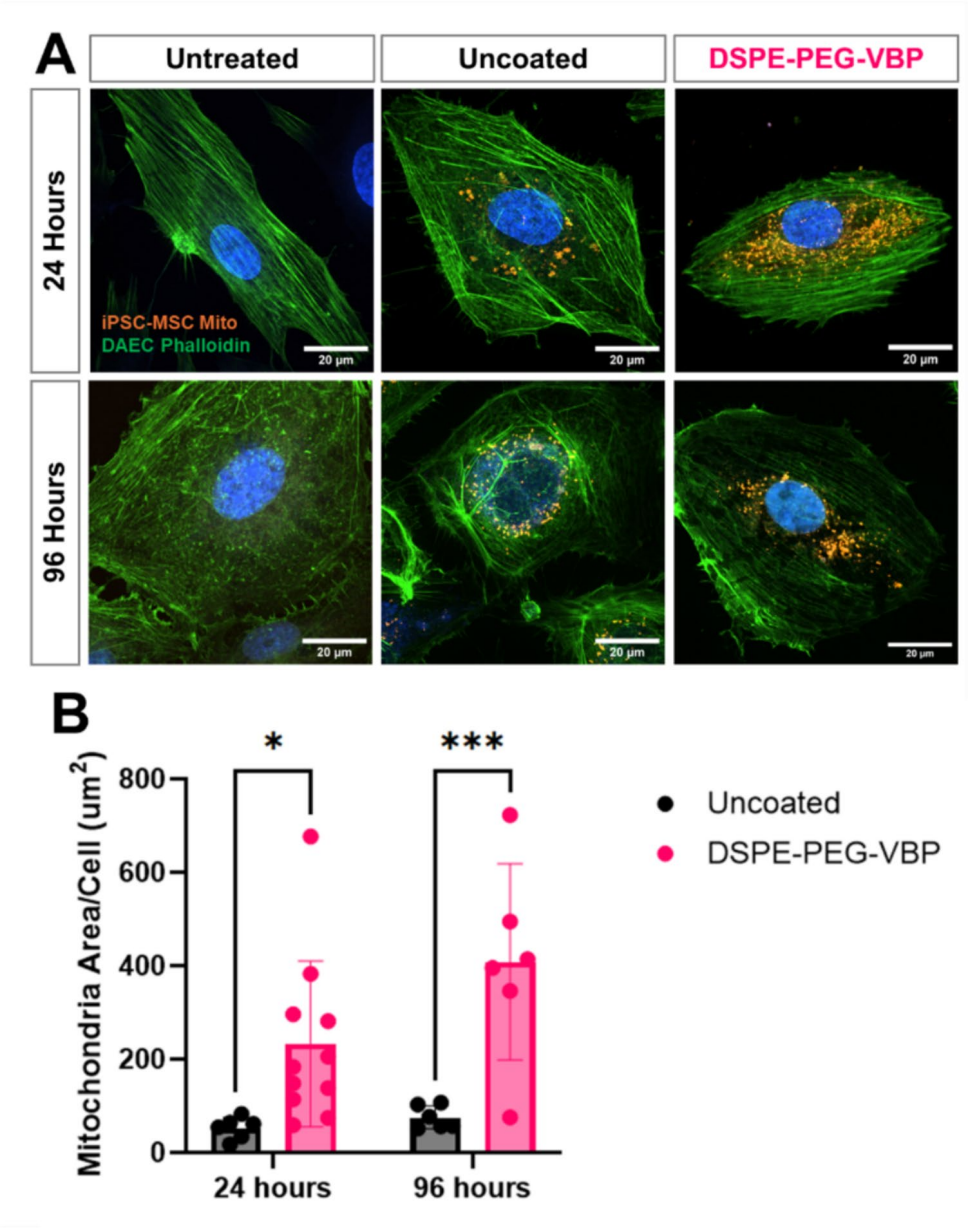
Endothelial uptake of engineered mitochondria. **A** Confocal microscopy of DAECs (Phalloidin-FITC) incubated with uncoated and DSPE-PEG-VBP MSC-derived mitochondria (MitoTracker Red) at 24 h and 96 h. Scale bar = 20 µm. **B** Quantification of mitochondrial fluorescence signals in recipient cells treated with coated mitochondria compared to uncoated mitochondria at both 24 and 96 h (*p < 0.05, ***p < 0.001, Two way ANOVA).

To assess the impact of surface engineering on mitochondrial uptake, human diabetic aortic endothelial cells (DAECs) were treated with MitoTracker Red–labeled mitochondria derived from iPSC-MSCs, either uncoated or coated with DSPE-PEG-VBP. Confocal imaging at 24 h post-treatment revealed intracellular localization of exogenous mitochondria in both groups, with notably more diffuse cytoplasmic distribution in the coated group (Fig. 4A). Quantitative analysis of fluorescence intensity per cell demonstrated significantly greater mitochondrial uptake in the DSPE-PEG–VBP–coated group compared to uncoated controls at both 24 and 96 h (Fig. 4B). No significant differences in uptake were observed between time points within each group, suggesting that the enhanced uptake conferred by surface functionalization is maintained over time. To further dissect the contributions of the lipid-polymer versus the targeting peptide, we conducted a follow-up study including an additional group treated with mitochondria coated with DSPE-PEG lacking peptide conjugation. DSPE-PEG coating alone enhanced mitochondrial uptake to a degree comparable to DSPE-PEG-VBP–coated mitochondria (Supplemental Fig. S2). These findings suggest that the lipid-polymer coating primarily drives the improved cellular uptake, while the targeting peptide may play a role in selective binding that warrants further investigation.

### Surface-Engineered Mitochondria Exhibit Sustained Colocalization and Morphological Remodeling in Endothelial Cells

**Fig. 5:**
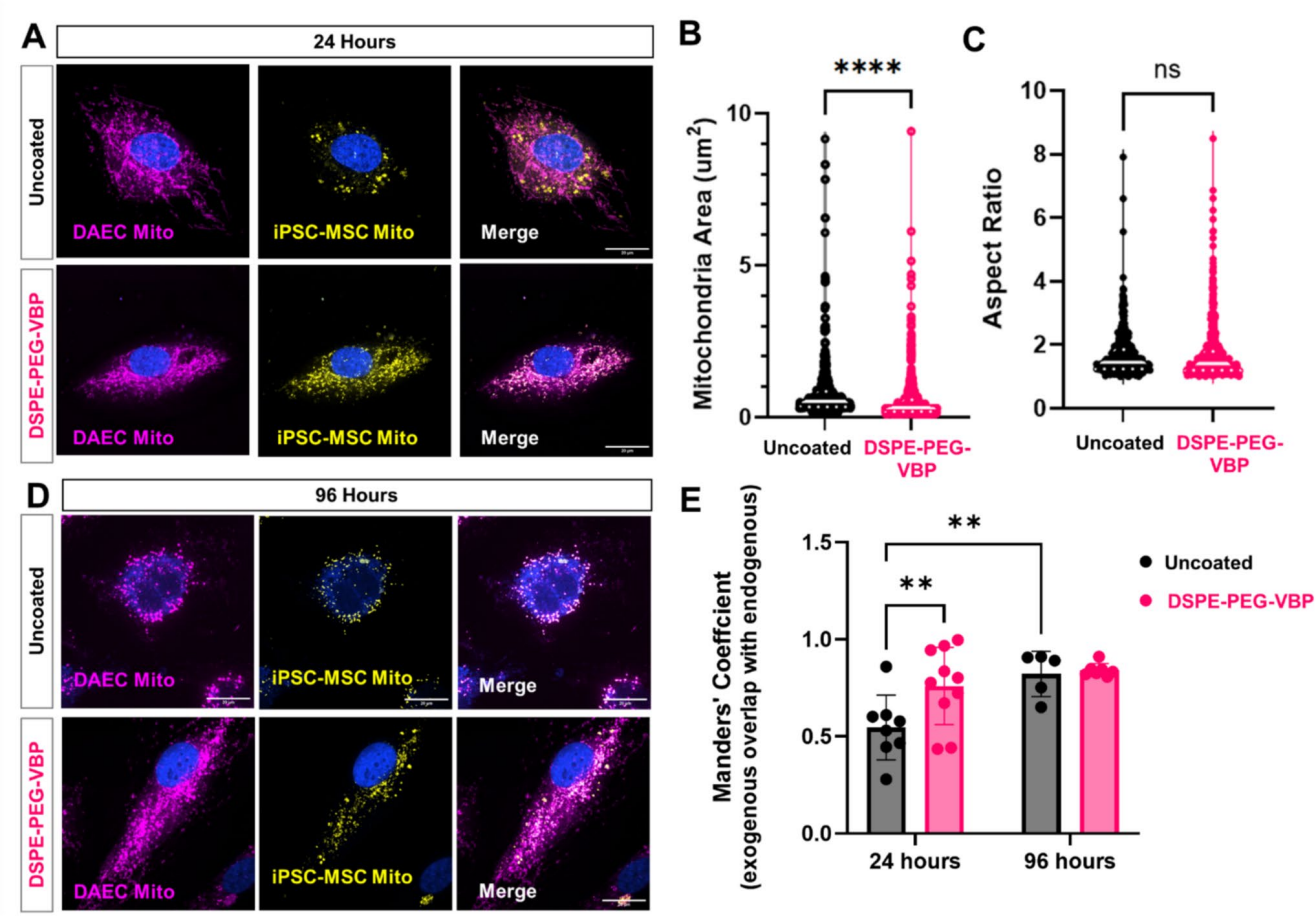
Exogenous mitochondria colocalization and morphological assessment. **A** Confocal microscopy of internalized endogenous (purple) vs. exogenous (yellow) mitochondria in DAECs at 24 h. Scale bar = 20 µm. **B** Size distribution of exogenous mitochondria at 24 h. **C** Aspect ratio of exogenous mitochondria at 24 h. Data represents mean ± SD from *n* = 510 mitochondria for uncoated and n = 619 mitochondria from 6 different images in each group. *p < 0.05 by unpaired two-tailed t-test. **D** Confocal microscopy of internalized endogenous (purple) vs. exogenous (yellow) mitochondria in DAECs at 96 h. Scale bar = 20 µm. **E** Quantification of colocalization between exogenous and endogenous mitochondria using Mander’s coefficient at 24 and 96 h. Data represents mean ± SD from *n* = 5-10 independent high power images. (**p < 0.01, Two way ANOVA.**)**

To further investigate the intracellular fate of exogenous mitochondria, we also labeled the endogenous mitochondria in DAECs with MitoTracker Deep Red, allowing for simultaneous visualization and quantitative assessment of colocalization with the exogenous mitochondria (Fig. 5). At 24 h, uncoated exogenous mitochondria appeared as discrete, spherical structures with minimal overlap with the endogenous mitochondrial network. In contrast, DSPE-PEG–peptide coated mitochondria were more diffusely distributed throughout the cytoplasm and exhibited greater visual colocalization with the endogenous network (Fig. 5A). Image-based particle size analysis revealed significantly smaller average area of internalized mitochondria in the DSPE-PEG–VBP group compared to uncoated controls (Fig. 5B), consistent with the reduced diameter observed after surface modification (Fig. 3B). However, analysis of mitochondrial aspect ratio did not show significant differences between groups at this time point (Fig. 5C). By 96 h, both groups showed improved co-localization of signals of endogenous and exogenous mitochondria (Fig. 5D). Quantitative analysis using Mander’s coefficient confirmed significantly higher colocalization in the DSPE-PEG–VBP group at 24 h. Although both groups exhibited increased colocalization over time, the difference between groups was no longer statistically significant at 96 h (Fig. 5E). These findings suggest that surface engineering facilitates early intracellular integration and retention of exogenous mitochondria and promotes alignment with the host mitochondrial network over time.

### Surface Engineering of Mitochondria Prolongs Functional Recovery in Endothelial Cells

**Fig. 6:**
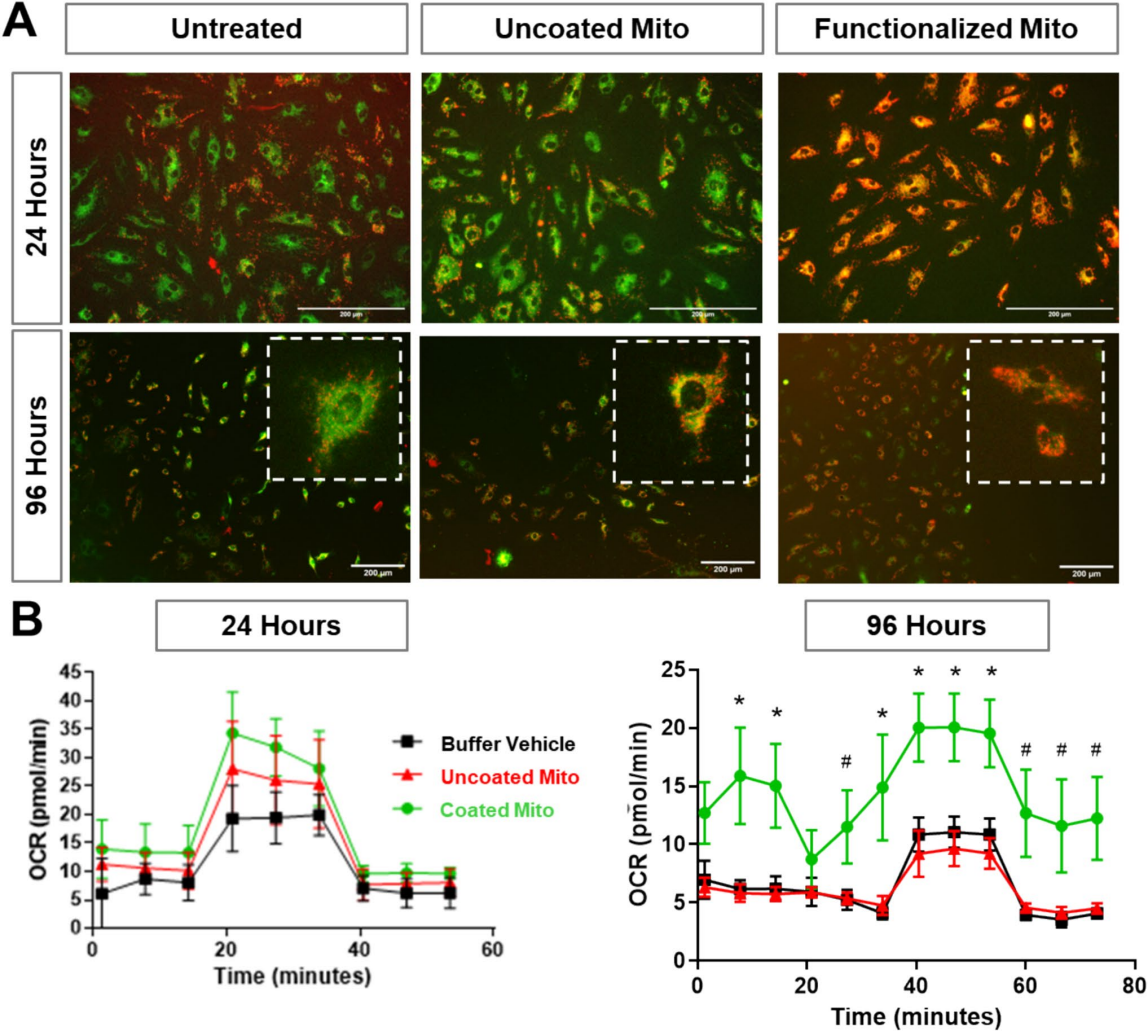
Functional assessment of DAECs after mitochondria uptake. **A** JC-1 staining of mitochondrial membrane potential in DAECs. Red: healthy polarized mitochondria; Green: dysfunctional depolarized mitochondria. **B** Seahorse analysis of OCR in DAECs treated with mitochondria at 24 and 96 h demonstrating improved respiratory function after engineered mitochondria transplantation (* denotes p < 0.05 Uncoated Mito vs. Coated Mito; # denotes p < 0.05 Buffer vs. Coated Mito using Kruskal-Wallis test with Dunn’ multiple comparisons test; n = 4; N = 1).

To evaluate the functional impact of mitochondrial delivery, we assessed mitochondrial membrane potential and bioenergetic function in DAECs following treatment with uncoated or coated MSC-derived mitochondria. Untreated DAECs served as a control group, representing the baseline mitochondrial dysfunction commonly observed in diabetes. JC-1 staining was used to assess mitochondrial membrane potential, where a shift from green (depolarized, dysfunctional mitochondria) to red (polarized, functional mitochondria) indicates improved mitochondrial health (Fig. 6A). In untreated DAECs, the predominant green signal reflected severe mitochondrial depolarization, consistent with diabetic endothelial dysfunction. Treatment with uncoated mitochondria induced a partial shift towards red fluorescence, suggesting an improvement in mitochondrial membrane potential. Notably, coated mitochondria resulted in a more pronounced shift towards red signals, indicating a greater restoration of mitochondrial membrane potential. To further assess mitochondrial function, we performed Seahorse extracellular flux analysis to measure oxygen consumption rates (OCR) as a readout of bioenergetic capacity (Fig. 6B). At 24 h post-treatment, both coated and uncoated mitochondria improved OCR in DAECs, albeit nonsignficantly, reflecting enhanced mitochondrial respiration. However, by 96 h, OCR levels in cells treated with uncoated mitochondria declined, whereas cells treated with DSPE-PEG mitochondria maintained significantly higher OCR levels, suggesting prolonged mitochondrial functional improvement with polymer coating. These results demonstrate that while both surface engineered and uncoated mitochondria transiently improve mitochondrial function in endothelial cells, DSPE-PEG polymer coating enhances the therapeutic effect and sustains functional benefits over time. This prolonged mitochondrial recovery may contribute to long-term endothelial protection and improved vascular health.

## Discussion

The potential of mitochondrial transplantation as a therapeutic strategy has been increasingly recognized, with recent studies demonstrating its role in vascular regeneration and cellular repair [5, 32]. For example, Lin et al. recently showed that mitochondrial transfer from MSCs enhances endothelial cell engraftment and vascular regeneration in ischemic injury models [5]. While these findings highlight the promise of mitochondrial therapy, several challenges remain for its clinical translation, including lack of targeting specificity, low cellular uptake efficiency, and compromised mitochondrial integrity during delivery. Our study addresses several limitations of conventional mitochondrial transplantation by introducing a surface-engineered mitochondrial delivery platform. Specifically, we developed a method to functionalize mitochondria surface with target-binding peptides using DSPE-PEG which also enhances uptake within dysfunctional endothelial cells. By leveraging surface functionalization, our approach improves mitochondrial delivery efficiency and promotes association with the host mitochondrial network, offering a promising strategy to advance cell-free mitochondrial therapy for endothelial dysfunction.

Several studies have explored polysaccharide-based coatings, such as dextran, to enhance mitochondrial stability, uptake, and therapeutic potential. For example, Wu et al. demonstrated that dextran-triphenylphosphonium (Dextran-TPP) functionalization of isolated mitochondria improved mitochondrial protection and cellular internalization compared to uncoated mitochondria, leading to metabolic benefits in various disease models, including cardiovascular and cancer cells [33]. A subsequent study from the same group showed that intravenous delivery of Dextran-TPP functionalized mitochondria reduced atherosclerotic plaque burden by targeting and reprogramming plaque macrophages, shifting their metabolism toward oxidative phosphorylation and reducing inflammation [32]. While these findings underscore the promise of polymer-assisted mitochondrial delivery, several challenges remain, including limited target specificity, variable mitochondrial retention, and uncertain long-term functional integration into recipient cells. Our approach builds upon these advances by introducing a bioactive lipid based polymer-functionalization strategy to enable targeting to dysfunctional endothelium. Unlike Dextran-TPP, which relies on passive accumulation, our DSPE-PEG-peptide functionalized mitochondria were engineered to interact with dysfunctional endothelium via VCAM-1 and subendothelial collagen interactions, providing targeting potential for disease-specific delivery in future applications. However, this in vitro study, conducted with a single endothelial cell type under static conditions, does not permit evaluation of peptide-mediated, target-specific mitochondrial delivery via CBP or VBP. To rigorously validate the targeting mechanism and determine selective binding, future studies should incorporate competitive inhibition assays, scrambled peptide controls, and co-culture systems with multiple vascular cell types.

Interestingly, we observed that DSPE-PEG surface functionalization alone was sufficient to significantly enhance mitochondrial uptake by endothelial cells, independent of conjugated targeting peptides (Fig. 4, Supplemental Fig. S2). While DSPE-PEG is often used as a linker to attach ligands or peptides, its amphiphilic structure and ability to insert into lipid membranes may facilitate enhanced interactions with the cell surface. The PEGylated surface may also reduce aggregation and improve colloidal stability, leading to increased bioavailability of exogenous mitochondria near the plasma membrane. Furthermore, the reduction in negative surface charge following DSPE-PEG coating, as observed in our zeta potential measurements (Fig. 3C), may decrease electrostatic repulsion between the negatively charged mitochondrial membrane and the endothelial glycocalyx, promoting uptake. These nonspecific biophysical effects of DSPE-PEG coating likely contribute to the increased internalization observed in our in vitro system, and should be considered when interpreting peptide-specific targeting effects. Future studies using additional controls and physiologically relevant models will be essential to decouple the contribution of biophysical surface modification from receptor-mediated targeting.

Additionally, we observed that the elevated internalization of engineered mitochondria persisted over time (Fig. 5). One potential explanation for the enhanced accumulation of coated mitochondria is that DSPE-PEG surface modification reduces clearance by extracellular and intracellular degradation pathways. Uncoated mitochondria may be more susceptible to extracellular damage and lysosomal autophagy after internalization, leading to their rapid clearance and loss of functional integration [34]. The ability of isolated mitochondria to survive in the high Ca^2+^ milieu post-transplantation has been a subject of debate [35]. Polymer functionalization may act as a protective barrier, shielding mitochondria from extracellular damage, reducing their recognition as foreign entities, and modulating interactions with intracellular clearance mechanisms. Exogenous mitochondria particles are analogous to lipid nanoparticle delivery systems, having a lipid bilayer membrane. It is plausible that DSPE-PEG-Peptide surface functionalization impacts endosomal escape which is a major bottleneck in the success of LNP-based therapeutics [36]. Using super-resolution microscopy, Cowan et al. demonstrated that exogenous mitochondria are transported to the endosomes and lysosomes after uptake, with most escaping and becoming fused with endogenous network [37]. However, these experiments only monitored mitochondria fate over 4 h while our study looked on the timescale of days. Apart from intracellular trafficking, DSPE-PEG may also alter the uptake mechanism of exogenous mitochondria to enhance the number of mitochondria that are initially internalized [38].

Another explanation for the observed enhancement is changes in mitochondrial dynamics, particularly fusion, fission, and biogenesis processes, which regulate mitochondrial function and cellular adaptation to stress by remodeling the mitochondria network [39]. The confocal imaging in this study (Fig. 5) suggest that coated mitochondria integrate into the endogenous mitochondrial network more rapidly and maintain an elongated, fusion-like morphology over time, while uncoated mitochondria initially appear isolated and fragmented. In agreement, Lin et al. showed uncoated mitochondria were not integrated into the host network and instead promoted mitophagy [5]. This observation raises the hypothesis that surface engineering promotes mitochondrial fusion, which is associated with greater metabolic stability and enhanced oxidative phosphorylation capacity. However, an important consideration in interpreting our colocalization data is the use of MitoTracker for labeling exogenous mitochondria. While this dye effectively labels mitochondria upon isolation, it is dependent on membrane potential and can leach or redistribute within the cell over time, potentially leading to overestimation of colocalization with the host mitochondrial network. As such, although we observed increased overlap between exogenous and endogenous mitochondrial signals in cells treated with DSPE-PEG–peptide–functionalized mitochondria, these findings should be interpreted cautiously. Future studies using genetically encoded mitochondrial reporters or dual-labeling strategies will be required to more accurately distinguish true mitochondrial interaction and fate after delivery. Additionally, the expression of fusion and fission proteins should be investigated at both the gene and protein levels to determine any alterations in mitochondria dynamics.

Another open question is how surface engineering affects mitochondrial bioenergetics upon delivery. The prolonged functional benefits of DSPE-PEG mitochondria, as evidenced by sustained improvements in OCR, suggest that DSPE-PEG may help preserve mitochondrial integrity and ATP production. Surely, we showed that DSPE-PEG functionalization did not negatively affect mitochondria respiration prior to delivery and improved oxidative coupling (Fig. 3D), however whether this is maintained after transfer into the hostile extracellular environment is unclear. Future research should focus on measuring mitochondrial function post-transplantation and performing transcriptomic analysis to uncover molecular regulators of enhanced mitochondria function to inform the optimization of our delivery platform. The sustained improvement in host mitochondrial function following treatment with engineered mitochondria has important implications for endothelial health. By restoring mitochondrial membrane potential and respiratory capacity in recipient cells (Fig. 6), our platform has the potential to reverse these pathogenic mechanisms and support vascular repair. While the current study focused on validating mitochondrial delivery, uptake, and subsequent effects on cell respiration, measurements of endothelial-specific functions such as nitric oxide production, permeability, and inflammatory activation were deferred for future studies to ensure differences in uptake did not confound results. Ongoing work is now evaluating the impact of our platform on endothelial function under inflammatory and metabolically stressed conditions in both in vitro and in vivo models.

In addition to surface engineering, the source of mitochondria is a critical factor that influences the therapeutic potential, scalability, and translational feasibility of mitochondrial transplantation. In this study, we utilized iPSC-MSCs, leveraging their high proliferative capacity, controlled expansion, and consistent mitochondrial quality for mitochondria therapy. Alternative sources of mitochondria, including tissue-derived and other cell-derived mitochondria, have been explored for various therapeutic applications [40]. Skeletal muscle-derived mitochondria are particularly attractive due to their high bioenergetic capacity and yield, making them a viable choice for transplantation. For example, several clinical trials (NCT02851758 and NCT04998357) and preclinical studies [4, 7, 41, 42] have utilized autologous skeletal muscle mitochondria to minimize immune rejection risks in patients. However, the need for invasive muscle biopsies poses a limitation, particularly for critically ill patients or those with advanced disease. Additionally, autologous mitochondria may not be viable in patients with mitochondrial dysfunction or genetic mitochondrial disorders, necessitating allogeneic sources. Emerging alternatives include extracellular vesicles (EVs) enriched with mitochondria, which may provide a physiological delivery system that enhances stability, uptake, and functional integration [43, 44]. However, the therapeutic benefits of EVs remain difficult to attribute solely to mitochondria, as other cellular components within EVs may also contribute to the observed effects. Ultimately, the optimal mitochondrial source must balance invasiveness, compatibility, and therapeutic efficacy. Still, a key limitation of this study is that the donor cells used for mitochondrial isolation were obtained from a single healthy male donor, representing a biological replicate of one. Future studies comparing donor cells from multiple individuals, including both sexes and varied genetic backgrounds, as well as mitochondria derived from other tissue sources, will be important to validate the robustness and generalizability of our findings.

Another major limitation is that DAECs, while they provide a disease-relevant endothelial model, were used under baseline culture conditions without the addition of diabetogenic, inflammatory, or hemodynamic stressors, and no endothelial-specific functional assessments which are critical for determining the broader physiological impact of mitochondrial transplantation on vascular health were conducted. Lastly, although our results demonstrate enhanced mitochondrial uptake in static culture, the ability of DSPE-PEG-peptide functionalization to provide endothelial specificity in the complex vascular environment and under flow conditions remains unverified. To address this, in vivo studies in vascular injury models are ongoing to evaluate target specificity, biodistribution, and therapeutic efficacy in physiologically relevant conditions.

In summary, this study establishes a proof-of-concept for a mitochondria delivery strategy to enhance uptake, cytoplasmic retention, and host mitochondria function in endothelial cells. By leveraging surface engineering via DSPE-PEG–peptide functionalization, we establish a modular platform that preserves donor mitochondrial bioenergetics and promotes intracellular remodeling. Given that mitochondrial dysfunction underlies numerous chronic diseases, including cardiovascular disease, metabolic disorders, neurodegeneration, and aging-related vascular decline, this approach lays the groundwork for a broad spectrum of applications. However, critical questions regarding in vivo biodistribution, immune response, long-term persistence, and functional durability remain to be addressed. Future studies will focus on evaluating therapeutic efficacy and safety in disease relevant models, optimizing dosing strategies, and incorporating physiologically relevant endothelial stressors. As our understanding of mitochondrial dynamics and intercellular transfer mechanisms deepens, engineered mitochondrial delivery systems may one day contribute to targeted therapies for a wide range of mitochondrial and metabolic disorders.

## Supplementary Information

Below is the link to the electronic supplementary material.

Supplementary file1 (DOCX 1009 kb)

## Data Availability

The datasets generated and analyzed during this study are available from the corresponding author upon reasonable request.
