# Supplementary material for: Surface-Engineered Mitochondria with Targeting Potential for Endothelial Repair
Source: Cell Mol Bioeng. 2025 Aug 22;18(5):403–17. doi: 10.1007/s12195-025-00862-1 (PMC12579636; doi:10.1007/s12195-025-00862-1)
Supplement: Supplementary file 1 — Supplementary file1 (DOCX 1009 kb) [file 12195_2025_862_MOESM1_ESM.docx]

Supplementary Information


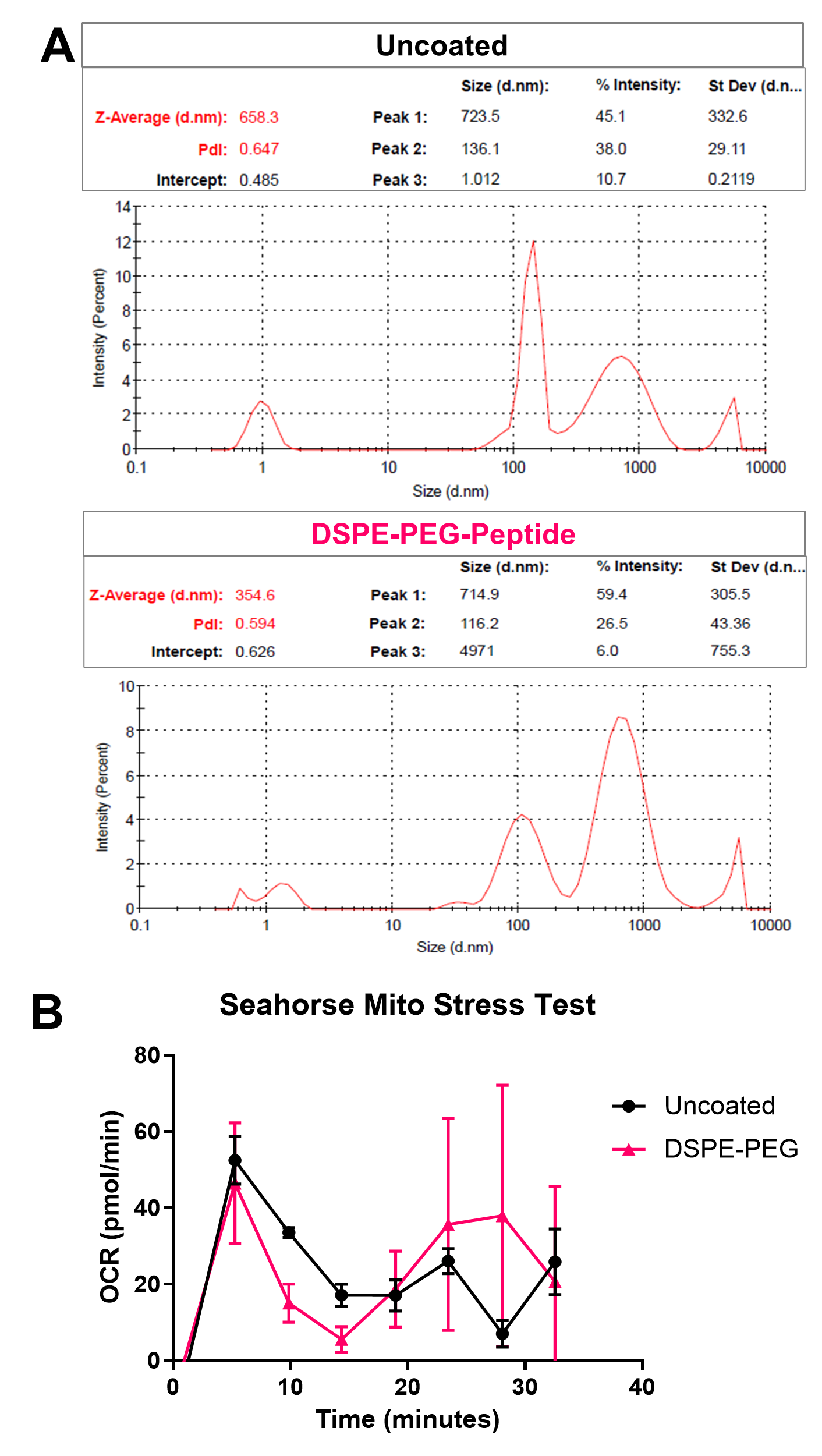


**Supplementary Figure S1: Characterization of DSPE-PEG Surface Engineering of Isolated Mitochondria.** (A) Representative size distribution plots of Uncoated (top) and DSPE-PEG-Peptide (bottom) mitochondria obtained from dynamic light scattering analysis. (B) Seahorse Mito Stress Test Extracellular Flux Analysis of Isolated mitochondria. Both groups showed similar OCR profiles with response to oligomycin, FCCP, and rotenone/antimycin at times 5 min, 14 min, and 23 min, respectively.

**
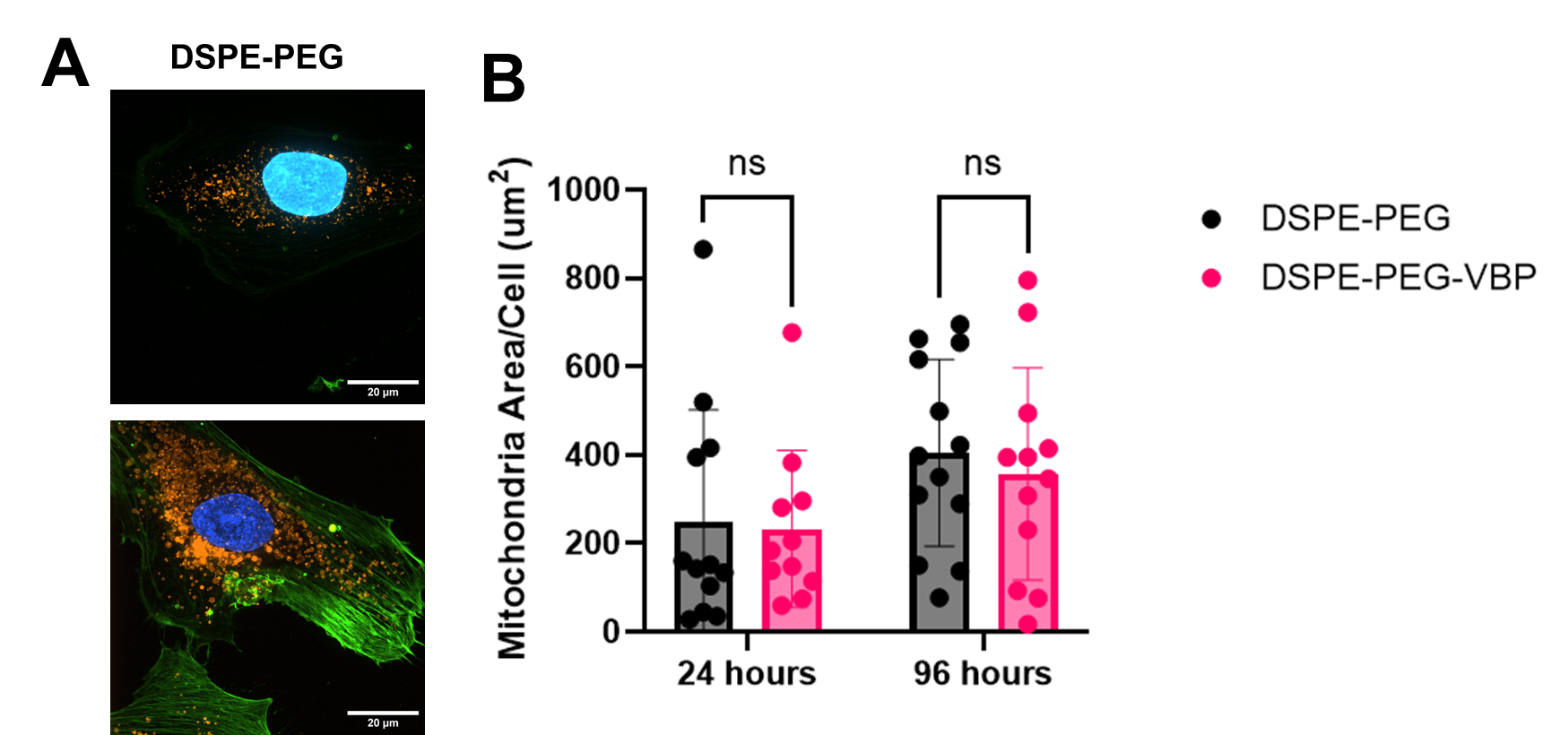
**

**Supplementary Figure 2: Effect of DSPE-PEG Surface Engineering on Mitochondria Uptake in Diabetic Endothelial Cells.** (A) Representative images of internalized Mitotracker labeled DSPE-PEG engineered mitochondria (orange) in DAECs at 24 hours (top) and 96 hours (bottom). (B) Quantification of uptake DSPE-PEG and DSPE-PEG-VBP engineered mitochondria. There was no significant differences in the area of internalized mitochondria between the groups at both time points, indicating that improved uptake stems from DSPE-PEG surface modification and not VBP (Data represents mean ± SD from n=3 replicates, Two way ANOVA).
